# Open and closed microfluidics for biosensing

**DOI:** 10.1016/j.mtbio.2024.101048

**Published:** 2024-04-04

**Authors:** Tianxin Ge, Wenxu Hu, Zilong Zhang, Xuexue He, Liqiu Wang, Xing Han, Zong Dai

**Affiliations:** a Guangdong Provincial Key Laboratory of Sensing Technology and Biomedical Instrument, School of Biomedical Engineering, Shenzhen Campus of Sun Yat-sen University, Sun Yat-sen University, No.66, Gongchang Road, Guangming District, Shenzhen, Guangdong, 518107, PR China; b Department of Mechanical Engineering, The Hong Kong Polytechnic University, 999077, Hong Kong, PR China

**Keywords:** Open microfluidics, Closed microfluidics, Biosensing, Immunoassay, Nucleic acid detection

## Abstract

Biosensing is vital for many areas like disease diagnosis, infectious disease prevention, and point-of-care monitoring. Microfluidics has been evidenced to be a powerful tool for biosensing via integrating biological detection processes into a palm-size chip. Based on the chip structure, microfluidics has two subdivision types: open microfluidics and closed microfluidics, whose operation methods would be diverse. In this review, we summarize fundamentals, liquid control methods, and applications of open and closed microfluidics separately, point out the bottlenecks, and propose potential directions of microfluidics-based biosensing.

## Graphical abstract

**Figure:**
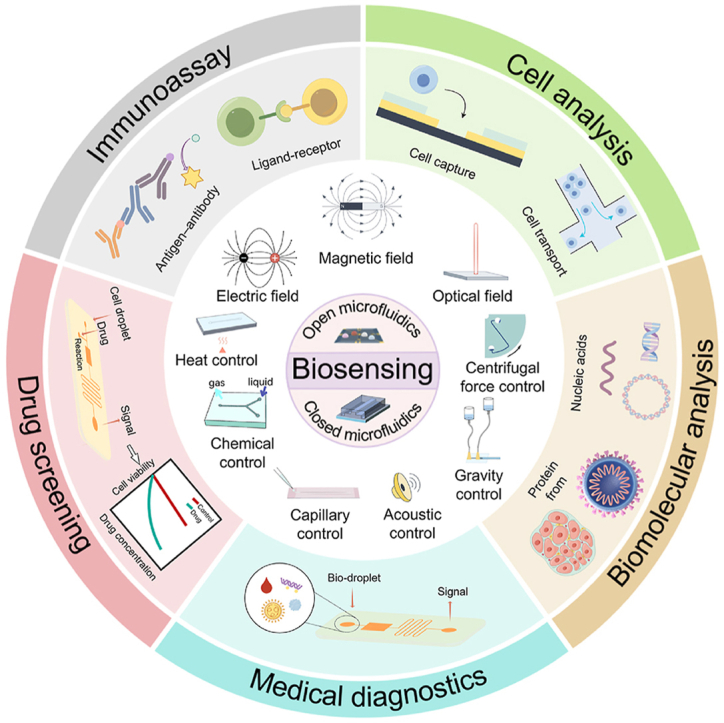

## 1 Introduction

Microfluidics has seen rapid advancements for decades with the advent of “micro total analysis system” in 1990, introducing an integrated analysis process microchip [[1], [2], [3], [4], [5], [6], [7], [8], [9]]. The ability of microfluidics to combine fundamental biological detection operating units, such as sample preparation, mixing, reaction, separation, and detection into a palm-sized chip, realizing various functions of conventional biology laboratories, has been shown to make it an effective tool for biosensing and to be one of the most cutting-edge scientific disciplines.

Microfluidics can be classified as open or closed depending on chip structure, and each kind demands corresponding liquid control approaches. Using a packed construction, closed microfluidics confines liquids inside channels and regulates their motion with a pump [10] or external physical fields [[11], [12], [13]]. Open microfluidics would allow liquids to contact air or another immiscible liquid [[14], [15], [16]], and droplets are often used for smaller sample volume (pL to μL droplets) and better maneuverability. It is of great importance to outline differences between the developed open and closed microfluidics, like liquid manipulation techniques, and biosensing applications since open and closed microfluidics should be carefully chosen and constructed for various biosensing applications.

In this review, we introduce open and closed microfluidics for biosensing in a systematical and distinct manner. For open microfluidics, we summarize fundamentals on wetting phenomena like contact angles, spreading parameter, and contact angle hysteresis of droplet on the surface, droplet manipulation methods (by electric, magnetic, or optical field), as well as applications in biosensing using open microfluidics, such as immunoassay, single-cell analysis, biomolecular analysis, drug screening, and medical diagnostics. For closed microfluidics, we summarize fundamentals in closed microfluidics like related dimensionless numbers and chip materials, liquid control methods, as well as applications like enzyme-linked immunosorbent assay and nucleic acids detection. The review is concluded with perspectives for further development of microfluidics-based biosensing and conclusions.

## 2 Biosensing via open microfluidics

### 2.1 Fundamentals

Open microfluidics typically performs the biological processes on open surfaces, making wetting phenomena vitally important. Wetting phenomena are pervasive in both nature and everyday life. For instance, a mercury droplet may spread out partially on a galvanized plate but not at all on a glass substrate; an aqueous droplet may form microscopic beads on a paraffin plate but spread out on glass.

Wetting refers to the ability of a liquid to spread or adhere on a solid surface to produce a uniform and continuous surface [17,18]. In a manner similar to the spreading of an aqueous droplet on a glass surface, the interface between a liquid droplet and a solid is driven by capillary forces towards equilibrium upon contact, achieving a state of spontaneous balance [19]. Hence, by altering the wetting behavior of droplets on a solid surface, it becomes feasible to exert precise and efficient droplet control on open surfaces. Within this framework, this section delves into fundamental principles of contact angle, spreading parameter, and contact angle hysteresis, with the aim of establishing a solid foundation for comprehending droplet manipulation techniques in subsequent discussions.

#### 2.1.1 Contact angle

**Fig. 1:**
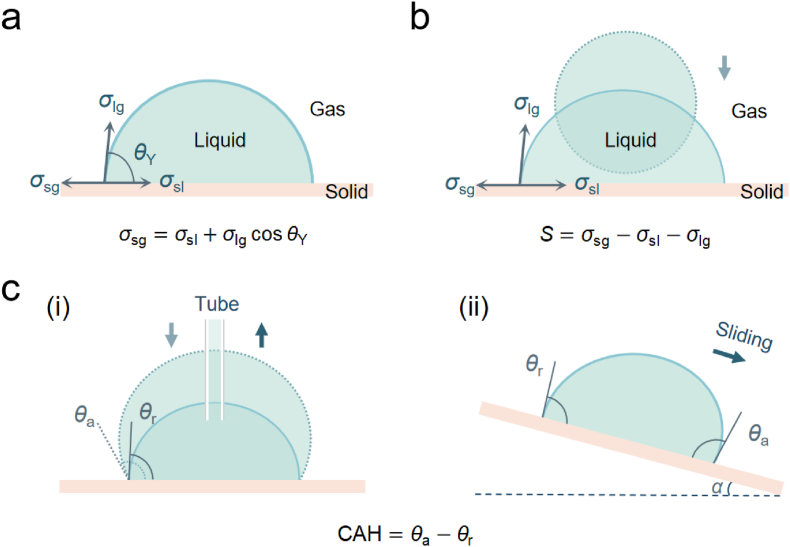
(a) interfacial tensions at three-phase contact line, droplet contact angle *θ*_Y_ on solid surface; (b) the spreading parameter on surfaces; (c) contact angle hysteresis (CAH), advancing angle *θ*_a_, receding angle *θ*_r_, i. on a horizontal plate and ii. on a tilting plate (critical sliding angle, *α*).

When a droplet is placed on a solid surface, droplet state depends on the interfacial tensions between the three phases of gas-liquid-solid (Fig. 1a). Interfacial tension refers to interaction force between surface molecules at the interface of two different substances. When an interface exists between a liquid and a solid, the angle between liquid surface and the solid surface is described as contact angle (CA, *θ*). Young's equation, which is suitable for the condition when a drop is placed on an ideal rigid, homogeneous, flat solid surface, can be expressed as [20].

(1) $$\sigma_{\text{sg}}=\sigma_{\text{sl}}+\sigma_{\mathrm{lg}}\;\cos\;\theta_{Y}\text{,}$$

where *σ*_sg_, *σ*_sl_, $\sigma_{\mathrm{lg}}$, $\theta_{Y}$ are solid-gas, solid-liquid, and liquid-gas interfacial tension, and Young's contact angle, respectively. Young's equation connects contact angle $\theta_{Y}$ with three interfacial tensions $\sigma_{\text{sg}}\text{,}$ *σ*_sl_, and $\sigma_{\mathrm{lg}}$. Despite the brevity of contact angle theoretical framework, the practical solid surfaces are naturally non-uniform with a certain degree of roughness [21], influenced by a multitude of factors like surface geometric morphology and surface chemical composition [22,23], posing a challenge in accurately determining Young's contact angle. As such, the measured contact angle is typically apparent contact angle. The apparent contact angle of a liquid can reflect wettability of a solid surface. For example, a water droplet deposits on glass with a contact angle of between 0° and 90°, namely hydrophilicity; a contrast case that an aqueous droplet deposits on Teflon with a contact angle of greater than 90° is named as hydrophobicity for the surface.

#### 2.1.2 Spreading parameter

Spreading parameter is closely related with contact angle, describing the wetting behaviors. Spreading parameter *S* can be described as^20^

(2) $$S=\sigma_{\text{sg}}-\sigma_{\text{sl}}-\sigma_{\mathrm{lg}}\text{,}$$

Spreading parameter can describe the tendency of a liquid phase to spread on solid phase (Fig. 1b). If *S* > 0, it indicates that the solid phase has a lower surface energy than the liquid phase, so the liquid wetting takes place spontaneously; in contrast, if *S* < 0, it suggests that wetting is incomplete. For complete wetting, *S* = 0. The spreading parameter can reflect the equilibrium wetting state of a liquid on a solid surface.

#### 2.1.3 Contact angle hysteresis

As shown in Fig. 1c i, a droplet sits on a horizontal plate. Upon injecting a small amount of liquid into the droplet, contact line remains stationary while the contact angle slightly increases. As more liquid is injected, the contact angle will continue increasing until it reaches a certain threshold (namely advancing angle *θ*_a_), at which point the contact line will begin to move forward. Similarly, if a small amount of liquid is continuously withdrawn from the droplet, the contact line will remain stationary until the contact angle decreases to a certain limit value, which is defined as the receding angle *θ*_r_. The difference between advancing and receding angles is defined as contact angle hysteresis (CAH), being important for the reflection of the degree of surface roughness and surface inhomogeneity.

(3) $$\text{CAH}=\theta_{a}-\theta_{r}\text{,}$$

The advancing and receding angles can also be determined by placing a droplet on an inclined plate (Fig. 1c ii). The forward edge of the droplet exhibits a larger contact angle than the backward edge. Nevertheless, the droplet will remain immobile until the tilted angle of the surface reaches a critical value that enables the droplet sliding. The critical sliding angle *α* is the slope angle at which a droplet begins to slide [24]. The advancing and receding angle is at forward and backward edge, respectively, when the droplet slides on the surface.

### 2.2 Droplet manipulation

Droplet manipulation is important for biological detection on open surface to undertake processes like liquid transport, mixing, and detection. Droplet manipulation methods can be divided into two types: active droplet manipulation and passive droplet manipulation according to whether external energy field is involved. Active manipulation usually refers to the manipulation of the liquid phase by external physical fields, such as thermal [25], acoustical [26,27], optical [28,29], magnetic [30,31], electric fields [[32], [33], [34], [35]], etc. This approach typically requires the specialized equipment or devices. In contrast, passive droplet manipulation primarily relies on the design of surfaces or materials that facilitate the controlled movement and positioning of droplets, such as processing asymmetry microstructures [36,37] or coatings to control movement of droplets on the surface [38]. This review focuses on the active droplet manipulation involving physical fields. The following section briefly introduce droplet manipulations by electric, magnetic, and optical field.

#### 2.2.1 Electric field

Droplet motion can be realized by asymmetry wettability [39,40]. By adjusting the interfacial tension between solid and liquid phases via applying an electric field, electrowetting technology provides a way to control droplets on solid surfaces. However, high electric pressure would cause water electrolysis. To address this, Berge et al. [41] proposed a thin insulating layer between the conducting liquid and the metal electrode, which led to the development of electrowetting on dielectric (EWOD). For EWOD, when a polar or conductive droplet is placed on an electrode coated with a dielectric layer, the potential difference between the droplet and the electrode leads to a reduction of contact angle. It causes asymmetric deformation at edges of the droplet, resulting in a pressure gradient inside the droplet. The flow inside the droplet will move toward the side with a smaller contact angle to reach the equilibrium, thus enabling the manipulation.

**Fig. 2:**
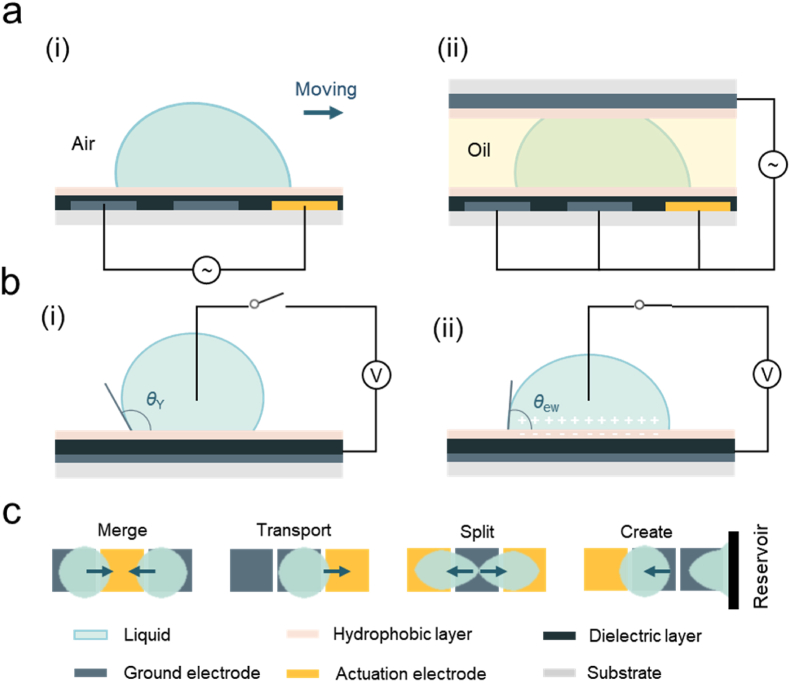
Diagrams of EWOD: (a) DMF device, i. one-plate (open) DMF device and ii. two-plate (confined) DMF device; (b) wetting cases, i. non-wetting and ii. wetting; (c) droplet manipulation: merge, transport, split, and create daughter droplets.

There are two types of existing EWOD-based systems: open system, in which droplets are allowed to freely rest on solid plates [42] (Fig. 2a i), and confined system, in which droplets are contained between two layers of plates [43] (Fig. 2a ii). In open system, droplets can contact with air, whereas in confined system, they typically encounter another immiscible liquid. The mechanism of both open and confined systems is similar, and following sections focus primarily on the open system due to its low flow resistance and higher freedom for droplets motion. The confined system is also classified as open microfluidics in this review instead of closed microfluidics replying on continuous flow.

A schematic diagram of a conventional EWOD is shown in Fig. 2, including a conductive droplet and an electrode covered by a dielectric and hydrophobic layer. After the voltage is applied, the conductive droplets' contact angle changes more noticeably. One of the most crucial qualities of an EWOD [34] is the range of CA fluctuation, which is governed by a variety of electrode and fluid system properties. After the applying of an electric field, the presence of an electric charge on the solid surface causes the aggregation of counter ions in the liquid and the formation of a double electric layer at the solid-liquid interface (Fig. 2b), which leads to a decrease in the effective solid-liquid interfacial tension. The contact angle of a droplet in equilibrium under the action of an electric field satisfies Young-Lippmann equation [44,45]:

(4) $$\cos\;\theta_{\text{ew}}=\cos\;\theta_{Y}+\frac{c_{H}{\left(V‐V_{\text{pzc}}\right)}^{2}}{2\sigma_{\mathrm{lg}}}\text{,}$$

where *θ*_ew_ is the contact angle at which a droplet reaches equilibrium with an electric field, *V*_pzc_ is potential of zero charge, *V* is external electric field intensity, *c*_H_ is double layer capacitance per unit area. The Young-Lippmann equation indicates that the contact angle decreases continuously with increasing electric field strength, which fits well on smooth hydrophobic surfaces and within a range of parameters. By applying an electrical potential between the droplet and the substrate below one side of the droplet to change its local contact angle, the EWOD-based digital microfluidics (DMF) induces aqueous droplet movement on a hydrophobic surface (Fig. 2c). This allows droplets to merge, transport, split, and create daughter droplets.

#### 2.2.2 Magnetic field

Despite the fact that EWOD-based digital microfluidics has garnered significant attentions, the induced electrolytic reactions and the influence to living organisms limit its applications [46]. Magnetic-field-controlled droplet manipulation can avoid the drawbacks. Hydrophobic low-friction substrates are used for droplet manipulation in both magnetic digital microfluidics (MDM) and EWOD-based digital microfluidics, while MDM substrate structure is simpler [31]. A flat surface covered in a hydrophobic substance (like Teflon AF) is sufficient for conventional MDM. Magnetic force can be regulated by magnetic particles or applied magnetic field, allowing for easy droplet manipulation, cost and device size reduction [47].

**Fig. 3:**
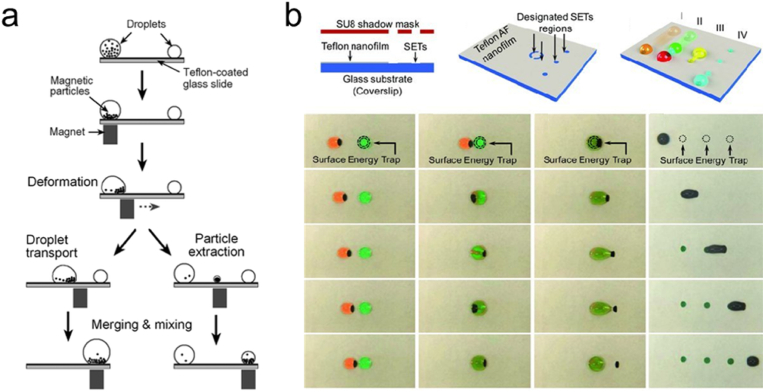
Magnetic digital microfluidics: (a) schematic set-up and fundamental operations in a magnet-actuated droplet system [46]. Copyright 2009, RSC; (b) droplet operations with SETs [50]. Copyright 2013, RSC.

Conventional magnet-actuated droplet system utilizes a magnetic field to manipulate droplets containing magnetizable particles. In addition to serving as droplet actuators, surfaces of magnetized particles can also be used as solid substrates for the uptake and separation of biomolecules [48,49]. However, the magnet-actuated droplet system is only capable of performing a few fundamental fluidic functions, such as sorting, mixing, and droplet transport (as shown in Fig. 3a). Thus, the applicability of MDM in complex assays, including liquid sample aliquoting, serial dilution, and droplet splitting for subsequent parallel and multiplex reactions [50] is significantly hindered.

To break through the limitation, Zhang et al. [50] developed a magnetic droplet manipulation platform based on surface energy traps, which are areas of high energy etched onto a low energy plating layer, where droplets were immobilized to facilitate droplet manipulation, enabling a full range of fluidic operations (Fig. 3b). The phenomenon occurs due to the surface's increased ability to attract and hold droplets. Subsequently, surface energy traps have been adopted by many MDM platforms to facilitate droplet manipulation as a key component required to achieve complex fluid manipulation [51,52].

#### 2.2.3 Optical field

Benefiting from its non-contact stimulus and tunable properties such as wavelength and power, light has become a crucial tool in microfluidics, providing excellent spatial (about micrometer) and temporal resolution (about microsecond or less) [[53], [54], [55]].

Photo-actuation of liquids converts light energy into liquid motion via direct optical forces or light-induced capillary forces, enabling precise manipulation of discrete pL to μL-sized droplets in applications [56]. Photonic momentum transfer can induce surface deformation of low-refractive-index media [[57], [58], [59], [60]], while optical tweezers (OT) can be used to capture and manipulate solid particles to drive droplets [61]. Both methods apply direct optical forces to achieve precise droplet manipulation. In addition, light could directly manipulate droplets through optical or light-induced capillary forces by generating a wettability gradient [62] or Marangoni effect [28,63]. Park et al. [64] integrated a photo-induced dielectrophoresis droplet manipulation platform based on a novel floating electrode optoelectronic tweezer to accomplish several important droplet manipulation functions, including droplet transfer, merging, mixing, as well as parallel, multifunctional, and multiple droplet manipulation.

**Fig. 4:**
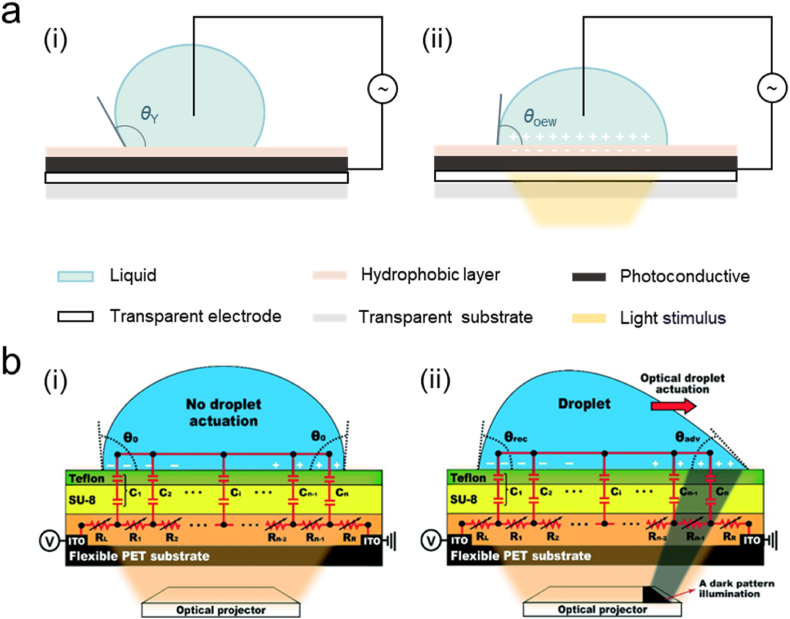
OEW digital microfluidics: (a) diagrams of OEW i. without light stimulus, ii. with light stimulus; (b) schematics of the SCOEW device simply fabricated by a spin-coating method on a flexible PET substrate and its equivalent circuit, i. droplet is placed, ii. droplet moves towards the dark pattern illuminated [69]. Copyright 2016, RSC.

Over past years, optoelectronic wetting (OEW) technology has garnered significant attentions. A novel approach for dynamic control of electrowetting on dielectric mechanisms using OEW was proposed by Chiou et al. [65], inserting a photoconductive layer between dielectric layer and electrode array, as shown in Fig. 4a. The conductivity of the photoreceptor improves under light by taking advantage of optical phenomena in semiconductors [66,67], resulting in a decrease in the impedance of its access to the circuit and a pressure difference inside the droplet on photoconductive and insulating layers. The droplet moves toward stimulus zone when a laser is focused on one side of the droplet to reduce the contact angle locally.

Based on this idea, Park et al. [68] developed single-sided continuous optoelectronic wetting (SCOEW) based on the OEW mechanism, which enables continuous droplet transport due to the featureless photoconductive layer of SCOEW. This advancement opens new possibilities for droplet manipulation on EWOD platforms. In order to impart photoconductive properties to optoelectronic wetting devices, previous studies have utilized amorphous silicon (a-Si) that is typically fabricated through high-temperature processes exceeding 300 °C. Jiang et al. [69] successfully prepared flexible SCOEW devices enabling light-driven 3D droplet manipulation via a polymer-based photoconductive material, titanium oxide phthalocyanine (TiOPc), overcoming thermal deformation of conventional flexible materials (such as polyethylene terephthalate and polyethylene naphthalate) during high-temperature processes (Fig. 4b).

### 2.3 Applications

Open microfluidics platforms have gained popularity in biosensing such as immunoassay, single-cell analysis, biomolecular analysis, drug screening, and medical diagnostics. This is because the platforms can manipulate complicated fluids with a great degree of precision and accuracy. The following section briefly sums up the works of the five fields (Table 1).

**Table 1:** Summary of open microfluidics.

Manipulation mode	Principle	Existing problem	Application	Ref.
Electric field	The droplet is placed on an electrode with a dielectric layer, generating a potential difference that causes asymmetric deformation and a pressure gradient	Limitation of electrode durability and stability; droplet drift and evaporation; high voltage requirement	Immunoassay	[[71], [72], [73], [74], [75], [76], [77], [78],^82^]
			Single-cell analysis	[88,89,91,92]
			Biomolecular analysis	[95,97,98]
			Drug screening	[[102], [103], [104]]
			Medical diagnostics	[[110], [111], [112], [113], [114]]
Magnetic field	Using a magnetic field to manipulate droplets containing magnetizable particles	Restriction of materials; limited manipulation range	Droplet manipulation technique	[[46], [47], [48]]
			Immunoassay	[51,81]
			Biomolecular analysis	[99]
Optical field	Photonic momentum transfer	Complex optical setups; inability for photosensitive samples	Droplet manipulation technique	[[57], [58], [59], [60]]
	Light-induced surface tension gradient			[28,62,63]
	Optical tweezers			[61]
	Optoelectronic wetting			[[65], [66], [67], [68], [69]]

#### 2.3.1 Immunoassay

The interaction between samples and antibodies is the foundation of immunoassays. By determining the concentration and activity of antigens in the sample matrix via technologies like fluorescence or absorption spectroscopy, biomolecules can be detected and quantified due to the high affinity and specificity of the binding between antigens and their corresponding antibodies. The reduced reagent usage and seamless integration with analytical methods are key advantages of DMF, making it an ideal platform for the development of advanced immunoassays [70]. Optical assays have been the mainstay of immunoassays, such as chemiluminescence [[71], [72], [73], [74], [75]], fluorescence [76,77], and Raman scattering [[78], [79], [80]] etc. By utilizing DMF in combination with these optical assays, new possibilities can be unlocked for advanced immunoassays with greater levels of sensitivity, specificity, and accuracy.

**Fig. 5:**
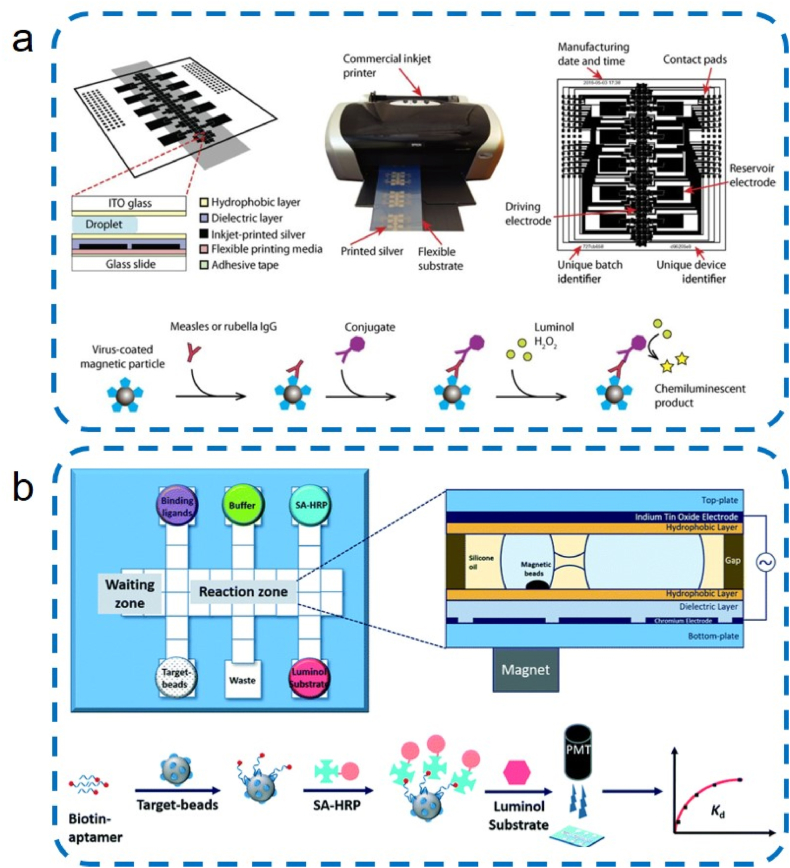
Digital microfluidic platform for immunoassays: (a) digital microfluidic cartridge and ELISA used for measles and rubella testing [72]. Copyright 2018, AAAS; (b) Schematic illustration of Auto-affitech [74]. Copyright 2020, RSC.

For chemiluminescence, Sista et al. [71] proposed a digital microfluidic platform for heterogeneous immunoassays that efficiently processed magnetic beads for the detection of human insulin and interleukin-6 (IL-6). This work demonstrated, for the first time, a heterogeneous sandwich immunoassay using magnetic beads on a droplet-based DMF platform. The retention of magnetic beads reached almost 100% even after multiple washes, providing a valuable guideline for subsequent magnet-actuated droplet system. The footprint of a single droplet was 1.5 mm. The success of this study has led to the emergence of MDM immunoassay platforms that can be used in combination with various assays [76,81]. Ng et al. [72] designed inkjet-printed digital microfluidic platforms for enzyme-linked immunosorbent assays (ELISAs) for field detection of measles virus and rubella virus-specific IgG in human whole blood acupuncture samples with 2.4 μL minimum volume covered two driving electrodes. (Fig. 5a). Guo et al. [74] designed an aptamer-protein system using digital microfluidics with a bidirectional magnetic separation method (Fig. 5b). The volumes of the unit droplet and the reservoir droplet were 1.7 μL and 7 μL, respectively.

**Fig. 6:**
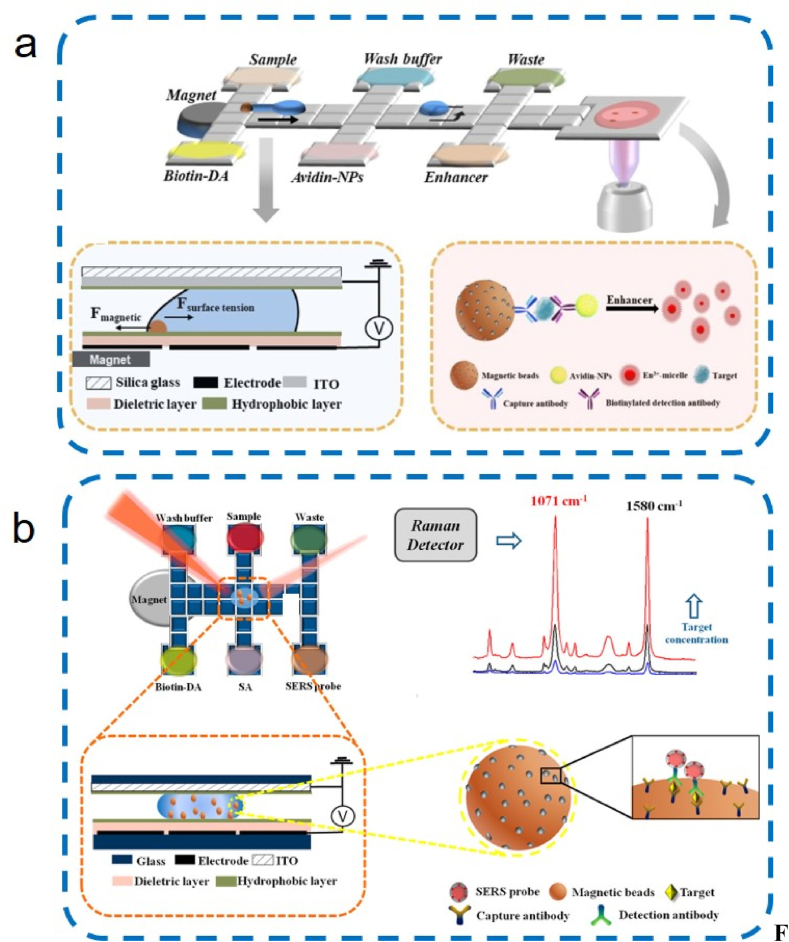
(a) Schematic of the dissolution-enhanced luminescence immunoassay with DMF [82]. Copyright 2023, ACS; (b) Schematic illustration of SERS-based immunoassay with digital microfluidics [78]. Copyright 2018, ACS.

For fluorescence, Vergauwe et al. [76] performed a non-competitive immunoassay for Immunoglobulin E (IgE) using 15-nm-diameter paramagnetic particles. The authors manipulated droplets containing fluorescently-labeled IgE aptamers and magnetic beads modified with anti-IgE to fuse with droplets containing unlabeled IgE and assayed the fused droplets. With a detection limit for IgE of 150 nM, this study demonstrates the potential of magnetic bead-based immunoassays on DMF platforms. Lu et al. [82] developed a dissolution-enhanced luminescence-enhanced digital microfluidics immunoassay, by using NaEuF_4_ NPs as nanoprobes to detect H5N1 hemagglutinin in human serum and saliva, on which the detection was achieved 1.16 pM with only 2 μL sample consumption (Fig. 6a).

For Raman scattering, Wang et al. [78] combined surface-enhanced Raman scattering (SERS) for the immunoassay of avian influenza virus H5N1 on a digital microfluidic platform with a detection limit of 74 pg mL^−1^ (Fig. 6b). Droplet size on this platform is influenced by variability in droplet partitioning and splitting processes.

In summary, the combination of optical assays with DMF platforms has enabled the development of various immunoassay techniques, including MDM, ELISA, and SERS, among others. These studies have demonstrated the potential of DMF platforms in detecting various analytes, including human insulin, IL-6, measles virus, rubella virus-specific IgG, IgE, and avian influenza virus H5N1.

#### 2.3.2 Single-cell analysis

Single-cell analysis [83] is a powerful technique for the study of individual living cells and can be applied in diverse fields such as cytology [12], immunocytochemistry [84], nucleic acid [85,86], and protein analysis [87,88]. Single-cell analysis provides insights into the cellular heterogeneity, differences between cells, and changes in different states of the same cell [89], leading to a better understanding of cellular processes. On a digital microfluidic platform, the exact isolation and analysis of single cells enhances the accuracy and precision of analysis, lowers interference and contamination between cells, and prevents sample loss during cell isolation and processing [85,90].

For cytology, Kumar et al. [89] integrated OT and DMF platforms to selectively capture Salmonella typhimurium using magnetic beads with 2.7 μL droplets. The OT was used to select magnetic beads with target bacteria based on their fluorescence expression for further localization, proliferation, and analysis. However, the potential damage to cells by the light tweezers remains to be studied. In another study, Zhai et al. [91] developed a DMF system with a 3D microstructure for single-cell isolation and long-term culture. They combined low-evaporation-temperature oils, surfactants, and low-voltage (36 V) for droplet transport to enable drug susceptibility testing. The DMF platform was used to detect droplet sizes of 1 mm. Using Cisplatin as a drug model, the system was used to investigate the drug sensitivity of MDA-MB-231 breast cancer cells and MCF-10A healthy breast cells. The test findings revealed a 10% inaccuracy in the control group, proving the system's accuracy and precision. However, the potential impact of low-voltage droplet transport on cell viability and drug susceptibility testing was not investigated in this study, requiring further investigation.

For protein analysis, Peng et al. [92] proposed an all-in-one DMF pipeline which provides a comprehensive and efficient solution for proteomic sample preparation. The MCF-7 and MDA-MB-231 breast cancer cell lines were used to assess the effectiveness of this method, and 973 proteins were discovered. This method's potential for therapeutic applications was further demonstrated by the analysis of human breast cancer tissue samples. This method's effective use on samples of human breast cancer tissue points to its potential for use in later clinical trials for cancer diagnosis and treatment. In conclusion, further reduction of cell damage is needed to successfully advance the DMF platform in single cell analysis.

#### 2.3.3 Biomolecular analysis

Biological molecule analysis involves the study of the structure, function, and interactions of various biomolecules, including nucleic acids, proteins, lipids, and carbohydrates, being essential for understanding biological processes and disease mechanisms.

For nucleic acids analysis, Shin et al. [93,94] pioneered the use of inkjet printing to prepare paper-based microfluidic chips and demonstrated multiplex detection of three biomarkers (glucose, dopamine, and uric acid) in human serum. Riley et al. [95] developed a digital real-time polymerase chain reaction (dqPCR) microfluidic assay to quantify internal DNA cargo from individual liposomes. The research utilized microfluidic digital PCR to examine DNA loading within liposomes, gauging their size, concentration, and morphology. Coelho et al. [96] designed a DMF platform for real-time amplification of ring-mediated isothermal amplification (LAMP) of DNA to monitor the cancer biomarker c-Myc using fluorescence microscopy, successfully amplifying 90 pg of target DNA (0.5 ng μL^−1^) in a short time. To achieve a volume of 1.62 μL per large droplet, nine small droplets of 180 nL each are required.

For disease markers, Shen et al. [97] constructed a universal multichannel immunosensor based on DMF thermal control chip with high sensitivity for detecting biomarkers of acute myocardial infarction, including Myo, CK-MB, and cTnI in human serum samples. Downstream electrode turned on and off to generate ∼1.7 μL droplet. The limit of detection (LoD) for the three biomarkers were 0.003, 0.30, and 10.50 ng mL^−1^, respectively. Haghayegh et al. [98] proposed the first self-powered microfluidic integrated electrochemical immunobiosensor, which realized the sensitive detection of SARS-CoV-2 N protein on the DMF platform. The biosensor successfully detected the protein analyte within the linear dynamic detection range of 10–1000 pg mL^−1^, with the LoD of 3.10 pg mL^−1^. Zhang et al. [99] constructed a fully integrated and automated MDM platform for Gram-negative bacilli, and although the sensitivity needs to be improved, it somewhat illustrates the platform's potential for epidemiological surveillance or other scenarios requiring relatively extensive Gram-negative bacilli screening.

These innovative approaches demonstrate the potentials of open microfluidics platforms for biological molecule analysis and their potential applications in clinical diagnostics and point-of-care testing (POCT).

#### 2.3.4 Drug screening

Drug screening is a process of testing and evaluating numerous chemical compounds to identify potential drug candidates that can be used to treat a specific disease or pathological condition [100]. Microfluidics is a powerful tool for cell-based drug screening [101], making it possible to perform high-throughput assessments of drug toxicity, efficacy, and metabolism. Here, we introduce cell-based screening applications via open microfluidics.

For cell-based drug screening, Bogojevic et al. [102] used a DMF platform to study cytotoxic effects by monitoring apoptosis. They generated dose-response curves of caspase-3 activity as a function of astrocystin concentration using both DMF and conventional techniques, and found a 33-fold reduction in reagent consumption with DMF. Au et al. [103] developed a microfluidic organoids for drug screening platform that uses digital microfluidics to create and analyze miniature hepatocyte-containing organoids on a micro-scale with a 0.63/1.36 μL droplet. This approach allows for automated drug screening of multiple candidates under different conditions simultaneously. Zhai et al. [104] developed a convenient single and multi-drug screening DMF technique to test the toxicity of two chemotherapeutics: cisplatin and epirubicin, towards MDA-MB-231 breast cancer cells and MCF-10A normal breast cells. This system allows for screening of drug concentrations up to four orders of magnitude with minimal sample consumption (∼0.6 μL). It also enables on-chip screening of single or multi-drugs. Ma et al. [105] developed 96-well microtiter plates to analyze drug response in primary tumor samples and cell lines. The drug response analysis of primary tumor samples showed good IC50 prediction.

These studies demonstrate the significant potential of open microfluidics platforms for drug testing and cell-based screening. DMF technology enables accurate analysis, less reagent usage, automated screening, and the assessment of drug reactions in various cell types and environments. These developments aid in the creation of effective and efficient drug screening techniques.

#### 2.3.5 Medical diagnostics

Medical diagnostics in modern medicine is focused on integrating early, prognostic, *in vitro*, and *in situ* diagnosis and treatment of diseases. *In vitro* diagnostics, including blood analysis, biomarker testing, genetic testing, and microbiological testing, are increasingly utilizing microfluidics technology [[106], [107], [108], [109]]. For blood analysis, Emani et al. [110] conducted enzyme function assays for antithrombin III, protein C, plasminogen, and factor VIII on a microfluidic chip. The limits of detection for the functional assay of the four blood coagulation factors were 12 % function, 5 % function, 17 % function, and 6 % function, respectively. Their findings suggest that small sample quantities can be employed for enzyme function testing using digital microfluidics technology. This method has multiple benefits, including lower costs and a smaller blood sample size (25 μL). Dixon et al. [111] developed a DMF platform for plasma separation from fingertip blood. The platform allows complex multi-step diagnostic analyses and can be easily operated with a single tap. The researchers demonstrated a 21-step rubella virus IgG immunoassay with a detection limit of 1.9 IU mL^−1^. Only 55 μL of whole blood were required for the process.

For biomarker testing, Sista et al. [112] developed a digital microfluidic platform to rapidly analyze acid α-glucosidase and acid α-galactosidase for Pompe and Fabry disorders (per 0.3 μL droplet). In order to achieve the required sample throughput for newborn screening laboratories, a disposable digital microfluidic cartridge was developed for multiplex analysis, being validated for Pompe, Fabry, Hunter, Gaucher, and Hurler [113]. Singh et al. [114] modified an open microfluidic platform authorized for newborn screening to analyze 12 enzyme reactions from a single dried blood spot punch. Their platform allows up to 1000 enzyme assays from a single 3.2 mm dried spot punch (100 μL extraction volume) with only 100 nL of sample needed for each experiment. These investigations show how beneficial open microfluidics is for blood analysis and biomarker testing.

## 3 Biosensing via closed microfluidics

We have summarized the fundamentals, droplet manipulation methods, and applications of open microfluidics. In the meanwhile, closed microfluidics is widely-used which consists of a network of multiple channels and performs the functions of chemical and/or biological laboratories [115,116]. Unlike open microfluidic chips [117], closed microfluidic chips require package of channel networks, which means fluid flow is surrounded by walls. The fluid movement in microfluidic chips generally follows the basic micro-fluid mechanics. Here, we briefly introduce fundamentals, liquid flow control methods, and applications in continuous flow microfluidics (Table 2).

**Table 2:** Summary of closed microfluidics.

Manipulation mode		Principle	Existing problem	Application	Ref.
Active methods	Electroosmosis	Solution generates electroosmotic flow under an electric field	Complicated devices	Cell transport	[157,162]
				Gene analysis	[218]
	Dielectrophoresis	The translational motion of neutral particles in non-uniform electric fields due to dielectric polarization	Complicated devices	Nanoparticle sorting	[164,165,169,170]
				Cell enrichment	[167,168]
	Acoustic waves	Sound waves cause pressure fluctuations and disturbances in fluids	External equipment (signal generation and amplifiers); low throughput	Single cell analysis	[171,172]
				Cell enrichment	[173]
	Centrifugal force	Fluid control by adjusting the speed or direction of the centrifuge	Centrifugal driven mechanism; bulky systems	Molecular diagnosis	[176]
				Enrichment of immune targets	[174,211]
				Perfusion cell cultures	[11]
	Heat methods	Thermosensitive materials undergo deformation when temperature changes	Limitation for temperature-sensitive biological samples	ELISA	[181]
				NA amplification	[178,219]
	Chemical methods	Convert chemical energy to fluid propulsion	Difficulty in precise control; byproduct generation	Small molecule detection	[188]
				Particles manipulation	[154,191]
				Heavy metal detection	[190]
	Optical methods	Photo-sensitive materials	Complex processing of optical materials	Liquid manipulation	[195]
				High-speed flows generation	[155,196]
Passive methods	Gravity methods	Gravitational potential energy	Inability for dynamic or pulsatile flow generation	Immunoassays	[197]
				NA detection	[198]
	Capillary methods	The capillary force that naturally rises or falls with liquids that are wetted or non-wetted with the pipe wall	Difficulty in precise control; long liquid filling time	Biomarkers detection	[134,200,201,204]
				ELISA	[199,200,202]

### 3.1 Fundamentals

#### 3.1.1 Dimensionless number

As early as 1977, Batchelor [118] referred to the flow problem of feature lengths ranging from 0.1 to 10 μm as “Microhydrodynamics”. The field of “microscale fluid mechanics” has emerged due to complex flow phenomena in microfluidic devices. Microfluidic control can be thought of as the manipulation of nanoliter-volume fluids because the needed liquid volume is so tiny at the microscale. This requires primarily the following dimensionless parameters, describing relative importance of the physical quantities and simplifying the necessary experiments to depict the systems (Table 3): Reynolds number (Re), Péclet number (*Pe*), Capillary number (*Ca*), Weber number (We).

**Table 3:** Dimensionless numbers.

Symbol	Name	Formula	Physical Significance	Equation
Re	Reynolds number	$\text{Re}=\frac{\rho \mu L}{\eta }$	The ratio of inertia force to viscous force	Eq. 5
*Pe*	Péclet number	$\text{Pe}=\frac{\mu L}{D}$	The ratio of convection to diffusion	Eq. 6
Ca	Capillary number	$Ca=\frac{\eta \mu }{\gamma }$	The ratio of viscous resistance to interfacial tension	Eq. 7
We	Weber number	$\text{We}=\frac{\rho \mu^{2}L}{\gamma }$	The ratio of inertial force to surface tension	Eq. 8

Péclet number (*Pe*) and Reynolds number (Re) are commonly used in the study of continuous transport phenomena, such as microfluidic mixing [119]. The physical meaning of *Pe* is the ratio of convection rate to diffusion rate, where diffusion rate is driven by a certain concentration gradient (Table 3, eq. (5)). In macro-fluid mechanics, Re is the basis for the judgement of flow characteristics, which represents the relative importance of inertia force to viscous force [8] (Table 3, eq. (6)). For example, in pipe flow, the flow with Reynolds number less than 2300 is laminar flow, while the flow with Re being 2300–4000 is transitional state, and turbulent flow is defined as the flow with Re > 4000. However, due to space limitations in microfluidic channels (related to characteristic length *L*), Re is typically between 10^−6^ and 10. Therefore, viscous stress dominates compared with fluid inertia, resulting in typical laminar flow [8]. In laminar flow, fluid mass transfer is mainly through diffusion, which is quite slow and inconducive to mixing.

(5) $$\frac{fi}{f\nu }=\frac{\rho \mu L}{\eta }\equiv \text{Re}$$

(6) $$\frac{v}{u}=\frac{\mu L}{D}\equiv \text{Pe}$$

where *ρ* is fluid density, *μ* is superficial velocity, *L* is characteristic length, and *η* is dynamic viscosity of the fluid, *D* is the mass diffusion coefficient, *v* represents convective rate, *u* means diffusion rate, *f*_i_ is inertial stress, and *f*_v_ is viscous stress (or internal friction force).

On the microscale, interfacial and viscous forces predominate when taking into account the control effect of surface force on body force [119]. Capillary number and Weber number become significant, representing the ratio of viscous resistance to interfacial tension and inertial force to surface tension effect, respectively [120] (Table 3, eqs. (7), (8))).

(7) $$\frac{f\nu }{f\gamma }=\frac{\eta \mu }{\gamma }\equiv Ca$$

(8) $$\frac{f_{i}}{f_{\gamma }}=\frac{\rho \mu^{2}L}{\gamma }\equiv \text{We}$$

where *γ* is surface tension, *f*_v_ is viscous stress, and *f*_γ_ is capillary pressure.

At the macroscale where Weber number is much greater than 1.0, the effect of surface tension can be ignored. The Weber number is applied in situations where incompatible fluids create interfaces, such as generating droplets in microfluidic channels. The smaller Weber number means the surface tension is more important. Many phenomena, such as capillary phenomena, soap bubbles generation, and surface tension waves, occur with low Weber number.

#### 3.1.2 Materials

Materials, chip size, shape, design, processing, and surface modification are all factors in the manufacture of microfluidic chips. The choice of materials is the most important factor to take into account since surface properties of materials are substantially magnified at the micro/nanoscales. Four concepts listed below serve as a synopsis of general principle to choose chip materials: (1) the materials must be chemically and biologically compatible to ensure the stability and dependability of the device; (2) the chip material should minimize interference with the detecting signal and have good optical characteristics; (3) the surface must be easily modifiable in order to improve separation, purification, and detection of biomacromolecules, such as through the catalysis of nucleic acid amplification; (4) manufacturing process must be affordable and simple.

**Fig. 7:**
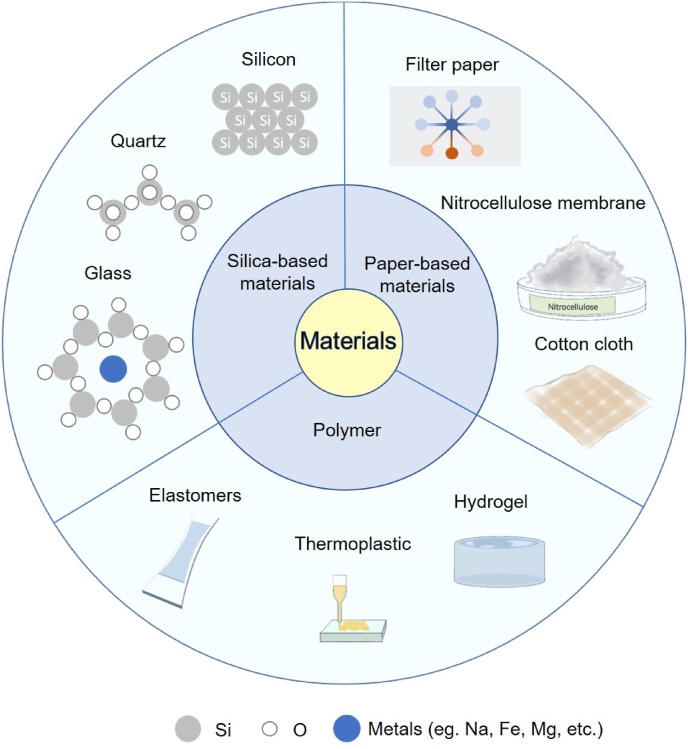
Substrate materials used in microfluidic chips.

Nowadays, three types of substrates most frequently employed in microfluidic chips are silica-based, polymer-based, and paper-based substrate (Fig. 7). Commonly-used silica-based materials are monocrystalline silicon, contain glass, and quartz. Polymers in lab-on-chip system varies, which can be divided into thermoplastic, elastomers, and hydrogel [121,122]. Paper-based microfluidic chips usually apply filter paper, nitrocellulose membrane, and cotton cloth as substrate materials. Here, we will introduce them in order.

##### 3.1.2.1 Silica-based materials

Silicon was first used in microfluidic chip for its chemical inertness and thermal stability [123,124]. Silicon wafers can be carved into highly precise two-dimensional graphics or three-dimensional structures by photolithography or etching. Li et al. [125] fabricated a family of digital polymerase chain reaction (dPCR) chips made of silicon with a unique layout design, which had 26,448–1,656,000 reaction wells. Besides, silicon is easy to be chemical modifies replying on the silanol group (-Si-OH). However, fragility, toxic chemicals (such the photoresist developer), and a relatively high cost of production are flaws shared by silicon and glass. Due to high elastic modulus, silicon has trouble in producing active valves and pumps [126]. As for optical performance, silicon cannot be penetrated by visible light though it is transparent to infrared, which makes typical fluorescence detection or fluid imaging a challenge for embedded structures [127]. Therefore, with the rise of glass and polymer materials, the application of silicon microfluidic chips has gradually decayed.

Glass receives widespread applications in biosensing due to its low background fluorescence and low non-specific adsorption. Thus, optofluidic detection can be performed using a glass microfluidic device. For example, Suzuki et al. [128] developed a borosilicate glass microfluidic platform for label-free chemical imaging flow cytometry by high-speed multicolor Stimulated Raman Scattering (SRS) microscopy. Moreover, ultra-thin glass sheet has a certain degree of flexibility and great potentials in flexible microfluidic platform, such as constructing micro-valves and/or pumps. Kazoe et al. [129] presented a 308 fL-valve chamber with a nanostructure fitting an arc-shape of deflected glass, and stably controlled an aqueous solution with a relatively fast response of 0.65 s. Nanofluids can be manipulated by this micro valve at 209 fL s^−1^ under a 100 kPa pressure in a 900 nm channel.

Similar to glass, quartz also owns good optical properties and electron-osmosis, and its surface reaction capabilities are conducive to surface modification [130]. Based on these superior properties of quartz, Lim et al. [131] combined 3D quartz nanohole arrays with microfluidic system and functionalized the arrays with streptavidin forming the STR-QNHA cell chip. Compared to opaque silicon nanowire-based platforms reported [132,133], the STR-QNHA platform possesses the advantage of transparency, which facilitates the use of fluorescence microscopy to observe images of cell capture and release [131]. However, quartz is relatively expensive, limiting its application to special testing needs, such as single molecule analysis and ultraviolet sensing. This limitation has driven the development of polymer chip materials, which are easy to process and shaping, low in material cost, suitable for mass production, and compatible with a wider range of biological applications.

##### 3.1.2.2 Polymer

Polydimethylsiloxane (PDMS), the most widely-used substance in lab-on-chip field, was first developed in the 1920s [134]. PDMS has a lot of benefits, including inexpensive microfabrication, UV light penetration over 250 nm, and breathability. Unger et al. [135] developed active PDMS-based microfluidic systems with multiple layers, allowing highly integration of valves. Owing to its gas permeability, PDMS chips have been employed in single cell analysis, such as cell culture, cell screening, and biochemical assays [136]. However, several studies have indicated drawbacks of PDMS. Since PDMS porous substrate's backbone is Si–O that has been overlaid by alkyl groups, some organic reagents are not compatible with it [126]. Second, the non-specific absorption of molecules reduces effective concentration of reagents [137].

Polymethyl methacrylate (PMMA) and polycarbonate (PC) were the early developments in thermoplastic microfluidic systems due to high optical transmission in the visible wavelengths and superior chemical compatibility [138]. Compared to PDMS, PMMA can achieve more stable covalent surface modification, especially after oxygen plasma treatment, whose surfaces can maintain hydrophilicity for a few years [126].

Paper-based microfluidic analytical devices (μPADs), or paper chips, use paper as the substrate material of the microfluidic chip owing to its highly porous structure [139]. Researchers use hydrophilic and hydrophobic modifications on the paper to accurately drain liquids through hydrophilic units. Matrix materials of μPADs mainly include filter paper [139], nitrocellulose (NC) membrane [140], and cotton fiber [141] etc. Early in 1980, lateral flow assay (LFA) emerged along with paper-based biosensors thriving, such as ELISA and vertical flow assay (VFA) [140]. Quantitative detection of immunoglobulin using paper-based ELISA in an instrumentless manner provide an alternative solution for portable diagnosis of numerous disease markers [142]. For example, Ma et al. [143] presented paper-based colorimetric ELISA to detect clenbuterol in milk, utilizing inherent properties of paper to embed antibodies. Samples and reagents are deposited onto the hydrophilic test zones confined by the hydrophobic barriers fabricated by wax printing and wax screen-printing.

Microcosmically, hydrogel is a three-dimensional grid structure composed of hydrophilic polymer chains, and the grid gap usually spans several nanometers to hundreds of nanometers [144,145]. Hydrogels have unique properties as the first biomaterial applied for human use [144], including high water content, high permeability, stable structure, cell-friendly, and high mechanical elasticity. Functional hydrogel structures can be integrated into microfluidic systems as active elements, including hydrogel bridges, biosensors, gradient generators, valves, and actuators as 3D cell culture environments [144]. In 2000, Beebe et al. [146] fabricated active hydrogel components performing both sensing and actuation functions inside microchannels *in situ* via direct photopatterning for autonomous flow control. Monomers and photoinitiators were injected into microchannels and irradiated through a photomask. This method could fabricate pH-responsive hydrogels of different chemical compositions directly in microfluidic channels. However, photo-polymerization involves toxic chemicals, complex rinsing steps, and bulky optical equipments. Rocca et al. [147] introduced a new method for *in situ* formation of hydrogels with a well-defined geometry in a sealed microfluidic chip by interfacial polymerization. Compared to traditional materials like glass and PDMS, hydrogel can be an excellent alternative material for chip substrate. Since hydrogel is non-toxic and compatible with cell adhesion, growth, and proliferation, it has been widely used for *vivo* microenvironment and cell-laden constructs [144,148].

### 3.2 Liquid control methods

The operation basis of lab-on-chip platforms is control technology of microfluids in microchannel network, which is the most essential feature of microfluidic chips different from dot-matrix microplates. The control and driving technology of microfluidics can be divided into active (light, sound, electricity, centrifugal, and magnetic force etc.) or passive methods (gravity, capillary force, and pressure etc.), depending on availability of external power source/field [149]. Here, we focus on well-developed driving methods, including active methods (electroosmosis, acoustic waves, centrifugal force, heat, chemical methods, optical methods, and dielectrophoresis) and passive methods (gravity and capillary-based methods).

#### 3.2.1 Active methods

Many active microfluidic control methods have been reported, including electric field-based methods [150], acoustic-wave-based methods [151], centrifugal-force-based methods [152], heat methods [153], chemical methods [154], and optical methods [155]. Active liquid flow control systems usually require mechanical or electrical actuations, and other external forces to perform precise continuous-flow control and large task scope [156]. Despite their complexity, they enable real-time adaptation of fluid flow rate and direction, offering great flexibility in fluid manipulation.

##### 3.2.1.1 Electroosmosis-based flow control methods

**Fig. 8:**
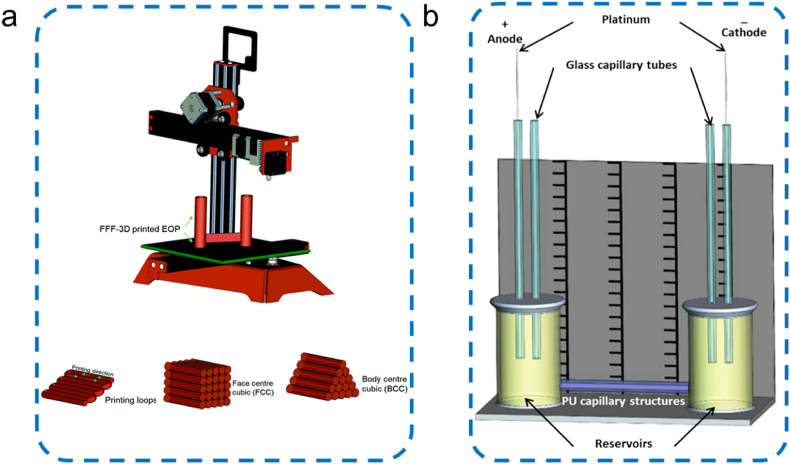
Thermoplastic polymers EOPs: (a) PLA capillary structures [161]. Copyright 2021, RCS; (b) PU capillary structures [163]. Copyright 2022, Springer.

As one of the most widely-used fluid control technologies, electroosmosis refers to the phenomenon that the liquid in microchannel moves along the inner wall of the channel in an overall direction under the action of an electric field [150]. The formation of electroosmosis needs an additional high-voltage power to regulate flow rate and direction. For example, electroosmotic pumps (EOPs) can create constant pulse-free flows and eliminate moving parts [157]. Based on these properties, EOP has been applied in various fields, such as chromatographic separation and drug delivery. However, the types of materials and manufacturing methods constrained the development of EOP in microfluidic systems to some extent. The materials for EOP mainly include glass [158], PDMS [159], and thermoplastic polymers [160], such as polyurethane (PU) [161], PMMA [162], and PC [160]. The processing and manufacturing methods of these materials usually include photolithography, etching, casting, bonding, hot embossing, and laser ablation to form microchannel structures. Wu et al. [161] created a novel fused filament fabrication (FFF) 3D printing technique as a cheap and accessible material extrusion approach for the 3D structure fabrication. To produce micro-capillary structures, polylactic acid (PLA) was deposited in longitudinal filament arrangements with in either “face-centre cubic” or “body-centre cubic” arrangements (Fig. 8a) [161]. The maximum flow rate of a single capillary EOP can reach 1.0 μL min^−1^. Except for PLA-based EOP, Wu et al. [163] also utilized thermally drawn thermoplastic PU capillary structures for microfluidic pumping (Fig. 8b). Using the fibre drawing technique, PU capillary fibres with internal diameters ranging from 73 to 200 μm was fabricated and used in single capillary and multi-capillary configurations [163].

##### 3.2.1.2 Dielectrophoresis-driven flow control methods

Dielectrophoretic (DEP) forces are produced when a non-unifrom electric field interacts with particle dipole moments [164]. The forces experienced by separated entities in an electric field are determined by distinct charges, potential gradients, and dielectric constants [164]. The electric field generated by alternating current (AC) or direct current (DC) induces polarization in charged or neutral particles. By varying the frequency of the applied electric field, the motion and arrangement of polarized particles can be manipulated [165]. This responsiveness is categorized as positive dielectrophoresis (p-DEP) or negative dielectrophoresis (n-DEP) [165]. Considering the varied electro-physiological properties of different particles, researchers have designed several electrode shapes (e.g. interdigitated electrodes (IDEs) [166], circular electrode [167], cylindrical electrodes [168] and planar tilted electrodes [169] etc.) to accommodate a broad range of biological detection targets. Zhang et al. [170] designed an IDE array at the bottom of the microchannel to induce n-DEP on particles, propelling them upward. Without altering the channel's dimensions or structure, the size of the target particle mixture can be changed by varying the voltage when an external dielectrophoretic force field is coupled with inertial forces. Using focus and sensing circular electrodes, Nguyen et al. [167] enriched circulating tumor cells (CTCs) from external electrodes to the central sensing zone. Due to the changes in impedance caused by the enrichment of cells on the sensing electrodes, they employed DEP-based impedance measurement (DEPIM) method. This approach facilitated the separation, concentration, and detection of rare cancer cells (A549 CTCs) in peripheral blood.

A large number of electrodes available for purchase are cyclic in shape. Compared to interdigitated and circular electrodes, cylindrical electrodes are easier to handle in large quantities. The commercially available cyclic electrodes have a simple structure and are easy to mass produce. They are usually embedded in microfluidic chips and integrated with detection components, such as dielectrics-based SERS sensors [168].

##### 3.2.1.3 Acoustic-wave-based flow control methods

At proper frequencies, acoustic waves basically wouldn't injure cells and biomolecules [171,172]. Thus, acoustic waves can be used in single cell analysis, such as single cells manipulations at micrometer-level precision [171]. For example, Liu et al. [173] created an acoustofluidic multi-well plate realizing simultaneous and consistent enrichment of biological cells in each well based on circular standing flexural waves. The device plate is comprised of an array of piezoelectric rings for the enrichment of micro/nanoscale cells in each well of the plate. The acoustofluidic multi-well plate is provided with simplicity, controllability, and biocompatibility, allowing it to become a powerful tool for acoustic-assisted in-plate tissue culture.

##### 3.2.1.4 Centrifugal-force-based flow control methods

**Fig. 9:**
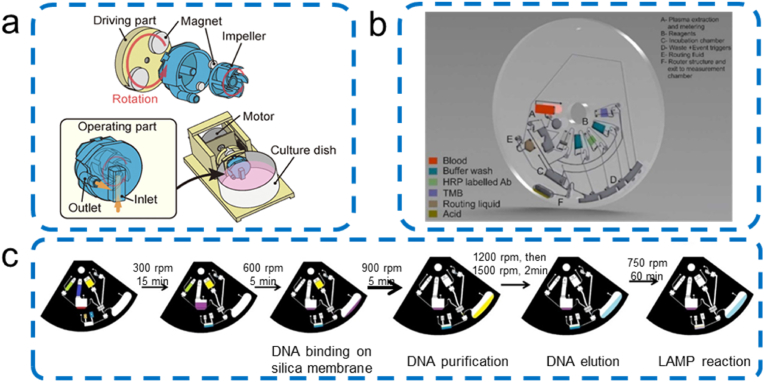
Scheme of centrifugal microfluidic LOAD platforms: (a) 3D-Printed Centrifugal Pump Driven by Magnetic Force [11]. Copyright 2022, Wiley; (b) lipophilic-membrane based router [174]. Copyright 2022, Elsevier; (c) integrated lab-on-a-disc for bacterial infection diagnosis [176]. Copyright 2022, Elsevier.

The two commonly-used micro-pumps are electric field-driven pumps (e.g. electroosmotic micro-pumps) and mechanical pumps (e.g. centrifugal pumps [174] and syringe pumps [175]). Centrifugal microfluidics, or lab-on-a-disk (LOAD) platforms, is a non-contact pumping technology [152]. However, a key issue of LOAD systems lies in establishing robust flow routing and valving on a rapidly rotating cartridge [174]. As shown in Fig. 9a, a 3D-printed centrifugal pump composed of the operating part and the driving part made by Jo et al. [11] is capable of precise flow rate control without physical contact, thanks to the permanent magnets on the motor's drive component. Due to its non-contact driving components, the pump is implemented in commercial petri dishes and tubes using a simple process [11]. However, despite its advantages, the system is difficult to implement reliable flow routing and full integration [11]. To optimize this challenge, Mishra et al. [174] utilized a novel solvent-selective membrane and a router structure for centrifugal stratification (Fig. 9b). This solvent selective lithium membrane can be used to construct solvent selective valves, as it can selectively dissolve in a non-aqueous, bioassay-compatible municipal liquid. Therefore, a novel solvent selective microfluidic routing mechanism shown in Fig. 9b has been designed to guide intermediate reaction products to alternative outlets to complete the reaction before optical measurement, thereby eliminating interference from optical detection. This mechanism, combined with event triggered flow control, can pave the way for large-scale integration and automation of rotational drive in centrifugal LOAD systems [174]. Loo et al. [176] created a sample-to-answer LOAD system implementing three main functions in predefined sequences, including DNA extraction, isothermal DNA amplification, and real-time signal detection for bacterial identification (Fig. 9c). The LOAD uses real-time loop mediated isothermal amplification (RT-LAMP) for molecular diagnosis of specific target bacterial DNA sequences, achieving a LoD of about 100 cfu mL^−1^ in sputum within 2 h.

##### 3.2.1.5 Heat-based flow control methods

Thermally-actuated microfluidics are typically powered by external power sources and can be integrated with other micro-manipulators, being beneficial for POC applications and large-scale automation. The materials for thermal-actuated microfluidic control usually rely on volumetric thermal expansion caused by temperature change for flow control, such as phase change materials [177] (e.g. paraffin and alkanes), and thermoresponsive gels. Heat is also involved and important in detections [178]. Here, we introduce thermally-actuated microfluidics with different actuator materials.

As micro-actuators, paraffin boasts the advantage of chemical inertness, rendering it highly biocompatible [179]. Moreover, paraffin typically offers large expansion rate (∼15%) upon transitioning from solid to liquid states [180], which inspired Masferrer et al. [181] to create a paraffin wax microvalve in microfluidic chips. When the wax barrier is heated and melts, the liquid (and gas) can flow through the valve. After the melted wax solidifies in the tunnel, it can block the fluids. However, paraffin possesses low thermal conductivity, resulting in high power consumption and long operating times, which makes it inefficient for a micro-actuator. Liu et al. [177] reported an inductive heating micro-actuator, employing high-conductivity materials (expanding graphite) and high-permeability materials (Ni particles) doped into PCM. Compared to traditional phase-change micro-actuators [182], it holds a crucial advantage: this micro-actuator generates heat within the paraffin composite material through inductive heating without any physical contact between the heater and the external power circuit. This implies that the micro-actuator needs no wires or electrodes connecting to the conductive PCM composite material. The structure and manufacturing process of this micro-actuator is simple.

Microfluidic control using thermo-responsive gels mostly relies on lower critical solution temperature (LCST) behavior of polymers [183], such as poly(N-isopropylacrylamide) (pNiPAAm) [184]. LCST phenomenon arises from temperature-dependent hydrogen bonding and hydrophobic interactions between polymers in aqueous solutions. For crosslinked hydrogels, when the temperature of the aqueous solution is above the LCST, polymer chains are hydrated and swollen. In contrary, the polymer chains drain water and attract one another as the temperature drops below the LCST, which causes the polymer volume to shrink [183]. Tudor et al. [185] took advantage of this phenomenon and synthesised a poly(ionic liquid) hydrogel named phosphonium-based crosslinked PIL tributylhexylphosphonium sulfopropylacrylate (PSPA). The hydrogel was integrated into a microfluidic device as low-cost temperature-controlled actuators, achieving multiple contraction and re-expansion in temperature cycles between 20 °C and 50 °C. Based on similar principles, Enferadi et al. [186] adapted the design from Quake's team [135] to create shape memory polymer (SMP) microvalves. These microvalves control fluid flow by inducing deflection in the SMP membrane through temperature variations.

##### 3.2.1.6 Chemical flow control methods

Chemical micro-pumps convert chemical energy into fluid propulsion using various stimulants. In comparison to other driving methods, they require no power source, making them more conducive to the overall miniaturization and portability of devices. The challenge in chemical micro-pumps lies in the need for precise control of reaction rates, concentrations, and other conditions to design programmable flows [154]. Various chemical reactions are employed to generate the desired gases or liquids, involving decomposition of H_2_O_2_ [187], pH changes [188], or specific catalysts [189]. The two main strategies used by chemical micropumps applying H_2_O_2_ decomposition are the noble metal dual electrode system [190] and H_2_O_2_ catalytic gas propulsion [191,192]. However, H_2_O_2_ solution is prone to decomposition at high temperatures, which is not conducive to long-term storage and transportation. In recent years, many chemical pumps adopt H_2_O_2_-free systems. Aishan et al.^189^demonstrated a chemical micro-pump driven by poly(NIPAAm-co-[Ru]-co-AMPS). Its mechanism relies on the Belousov-Zhabotinsky (BZ) reaction, wherein malonic acid is oxidized by an oxidizing agent. Then, the strong acid and metal catalyst substrate penetrate the polymer network, leading to redox oscillations, resulting in periodic changes in the size and color of the polymer gel. The repetitive expansion and contraction movements of the polymer gel rod serve as driving force for liquid pumping. As for the study of pH-based fluids control, Kong et al. [188] encapsulated Fe_3_O_4_ magnetic chains on a poly(acrylic acid (AA)-co-2-hydroxethyl methacrylate (HEMA)) substrate, forming pH-responsive cilia arrays. Changes in the local pH value cause the carboxyl group to undergo reversible ionization. These changes also affect the volume of hydrogel shell, which in turn influences the distance between particles (lattice constant) and the total length of the magnetic cilia, and finally the fluid pumping speed can be changed under the rotating magnetic field.

##### 3.2.1.7 Optical flow control methods

The transformation of light energy into mechanical energy for propulsion is the fundamental idea behind light-driven systems. Light is non-contact, dynamically reconfigurable, and easily focused onto minuscule measuring sites [193,194]. Some studies have utilized the photoresponsiveness of material surface microstructures or bulk structures deformation to manipulate fluids. Dradrach et al. [195] demonstrated a photo-peristaltic pump made by liquid crystal (LC) gels based on reversible strains in response to light. After light exposure, LC molecules in the nematic phase undergo nematic-isotropic phase transition, causing the contract of LC gel along the alignment direction and local protrusions on the strip surface at the laser spot. Most optical methods require noticeable visible moving mechanical components and doping of photosensitive materials. Wang et al. [196] combined the photoacoustic effect and acoustic streaming, utilizing pulsed laser to generate high-speed liquid flow. Specifically, when plasma nanoparticles are exposed to a pulsed laser, the photoacoustic effect produces ultrasonic waves due to the rapid volume expansion.

#### 3.2.2 Passive methods

Since active methods need multiple complex components like actuators, complex structure, and increased size, they are unfavorable for POCT. Passive microfluidic driving method is newly developed technology, which is widely adopted in POC diagnosis for its simple manufacture and low cost.

##### 3.2.2.1 Gravity-based flow control methods

Gravity-based flow control is a relatively simple and cheap method for microfluidic chips. The gravity force can generate hydrostatic pressure as a liquid driver simultaneously [106,149]. Since the flow is passively driven, the design can be extremely simple without an external actuator and power source. Reis et al. [197] presented a 10-bore microfluidic siphon as a portable ELISA system using only gravity instead of capillary force. Zai et al. [198] created a simple and minimizing gravity-driven cartridge while most traditional cartridge systems acquire auxiliary hardware via active pumping. To enhance gravity-driven fluid flow, the microfluidic cartridge contains an arc design at both ends of the chamber and hydrophilic modified channels [198]. Gravity-based flow control meets requirements of POCT for miniaturization and simplification without analytical performance reduction.

##### 3.2.2.2 Capillary-force-based flow control methods

In microchannel, there are many different methods for integrated flow control using capillary effect [199,200]. Negative capillary pressure can be used for fluid flow regulation and self-filling of capillary microfluidics [201]. This process often requires the rational design of capillary microvalves, micropumps and flow resistances to assemble into “capillary circuits” (CCs) [199,200]. However, so far, the automation of complex and repetitive liquid processing operations can almost only be achieved through software programs, professional operators, and bulky peripheral devices. To address the above issues, Yafia et al. [200] introduced a microfluidic chain reaction (MCR) as conditional, structurally-programmed propagation of capillary flow events without any external fluid control system. The MCR chip is based on capillary domino valves (CDVs) controlled liquid deterministic release, where the release of reservoir *n*+1 was triggered only when the reservoir *n* was emptied. An end-user can simply deposit a drop of sample solution at the inlet, where it is loaded with a capillary retention valve and lined with three stop valves (SVs), including one at the intersection of the functional connection and the main channel, and two with a dual function of retention burst valves (RBVs) [200]. However, MCR chip requires precise pipetting and relatively low concentration of surfactants [202]. High concentrations of surfactants can cause corner flow and bubble trapping, but 0.05% Tween 20 is essential for ELISA to prevent non-specific binding. Regarding these two issues, Parandakh et al. [202] fabricated a capillary equalization circuit (CAC) to perform automated ELISA on chip using MCR. They simplified chip loading and liquid transfer, allowing the solution to spontaneously fill measurement reservoirs, while CAC automatically discharged excess liquid from all reservoirs, forming aliquots with various volumes, allowing it to work under 0.05% Tween 20 conditions.

Another key method to achieve precise microfluidic control without external devices or professional operators is to use inkjet plotters to store picogram quantities of dry reagents with micrometer accuracy in microchannels. Based on self-coalescence phenomenon, Gökçe et al. [203] developed a self-coalescence module (SCM) to regulate inkjet-spotted reagents for *in situ* reactions or sequential delivery in microfluidic chips. This self-coalescence capillary flow phenomenon referred to the phenomenon where a confined liquid is forced to return to itself at a stretched gas-liquid interface in a microfluidic channel, in which case the reagent can be reconstructed with minimal dispersion [203]. Taking advantage of SCM chip, Salva et al. [204] performed a molecular beacons (MB) reaction and a clamped-hybridization chain reaction (C-HCR) in capillary-driven silicon microfluidic chips in about 2 min and using only about 3 μL of the sample. These results indicate that self-powered SCM microfluidic chips can provide a powerful platform for executing and researching complex reaction systems [203,204].

### 3.3 Applications

An ideal POC should be ASSURED, namely affordable, sensitive, specific, user-friendly, rapid and robust, equipment-free and deliverable to end users [205], as suggested by the World Health Organization (WHO). Microfluidic technology can provide a reliable platform for POCT, and here we introduce the applications of closed microfluidics, including ELISA and nucleic acids testing (NAT).

#### 3.3.1 Enzyme-linked immunosorbent assay

ELISA was founded and applied in immunoglobulin assay decades ago. It is now considered the “gold standard” for biological detection. The standard immunoassay procedures involve fixing the antigen or antibody to the surface of the well plate, adding sample antigen or antibody for incubation, obtaining the immune complex, separating it from the free component, and measuring the immune complex's concentration to determine how much antigen or antibody is present in the sample. In above processes, special markers (such as fluorescent molecules, enzymes, chemiluminescent chemicals, etc.) are labeled on the antigen or antibody during the processes; the use of enzyme markers during these processes is known as ELISA. Because of this, ELISA is composed of four basic components: a solid substrate, a recognition component, a signal amplification scheme, and a readout method [206]. Direct ELISA, sandwich ELISA, and competitive ELISA are three main types of ELISA models [207].

However, traditional ELISA has numerous limitations, like difficult manipulation, low sensitivity, and unsuitability for POCT. Thus, researchers combine ELISA with Micro Total Analysis System (μTAS) to achieve its complicated procedures automatically and use functional nanomaterials to modify substrates to enhance its sensitivity. Here, we focus on signal amplification, miniaturization, and automation strategies of ELISA for POCT.

In conventional ELISA, natural enzymes conjugates serve as both signal markers and catalysts. However, natural enzymes have many inherent disadvantages, such being difficult to separate and purify, having limited tolerance to acid and alkaline, and being unorthodox for storage. Natural enzymes have been replaced with nanomaterials having peroxidase activity known as nanozymes in recent years. Examples include Pt nanoparticles (PtNPs) [208] and Co–Fe nanohybrids [209]. By employing PtNPs to catalyze oxygen distance readings, Liu et al. [208] fabricated a fully-integrated ELISA-Chip that dramatically increased the sensitivity of ELISA. Excellent catalytic properties enable PtNPs to convert over one million H_2_O_2_ molecules into oxygen per second, amplifying signals by more than 10^10^ times within a few minutes of the reaction.

In addition, some metal organic frameworks (MOFs) [209], such as Fe-MOFs, have been reported to have enzymatic simulation properties. Based on this, Guo et al. [210] used Fe-MIL-88NH_2_ metal-organic framework (Fe-MOF) nanocubes and PtNPs to form Fe-MOF/PtNPs as peroxidase mimics to achieve the dual-signal enhancement. They used immunomagnetic nanoparticles (MNPs) and Fe-MOF/PtNPs to capture target bacteria, forming MNP bacteria Fe-MOF/PtNPs sandwich complexes. Fe-MOF/PtNPs decomposed H_2_O_2_ into O_2_, resulting in an increase in internal pressure that caused the preloaded water to react with the CaO powder and produce heat. Finally, the heat was measured using thermal sensor of a smartphone to quantitatively detect bacteria as low as 93 cfu mL^−1^ within 1 h. Due to their chemical stability and straightforward surface modification process, nanozymes have made significant advancements in the field of immunoassay. However, given that many of them have lower catalytic activity and worse substrate selectivity than natural enzymes, it is still challenging for them to replace natural enzymes as standard markers for ELISA analysis. Therefore, further constructing novel nanozymes with high substrate selectivity and catalytic efficiency is necessary.

Traditional ELISA's fluid operating program is time-consuming and requires a lot of reagents and samples, making it inappropriate for POCT. Programmable fluid flow management, avoiding unnecessary mixing to reduce antibody crosstalk, and removing potential operational errors are crucial for an on-chip ELISA [211]. To facilitate the miniaturization and automation of POCT ELISA, researchers developed sophisticated flow control technologies, particularly for the washing processes in small reactors. Using CLOCK (Control of Liquid Operation on Centrifugal HydroKinetics), which implements multi-step liquid injection and displacement, Okamoto et al. [212] developed an automatic and autonomous ELISA system. The device rotates at a stable frequency and controls injection time by adjusting the rotation rate. As a result, the interval time fluctuations of each step (antigen antibody reaction, substrate cleaning, and injection) is less than 5%. However, the centrifugal microfluidic device still requires complex device structures and manufacturing processes. He et al. [211] created composable microfluidic plates (cPlate) to control microfluidic reagents utilizing vertical movement without any external equipment. The cPlate is composed of a 96 well plate and a channel plate, in which flow control is implemented by the device structure, achieving portability and non-instrumentation, achieving portability and non-instrumentation.

#### 3.3.2 Nucleic acids detection

**Fig. 10:**
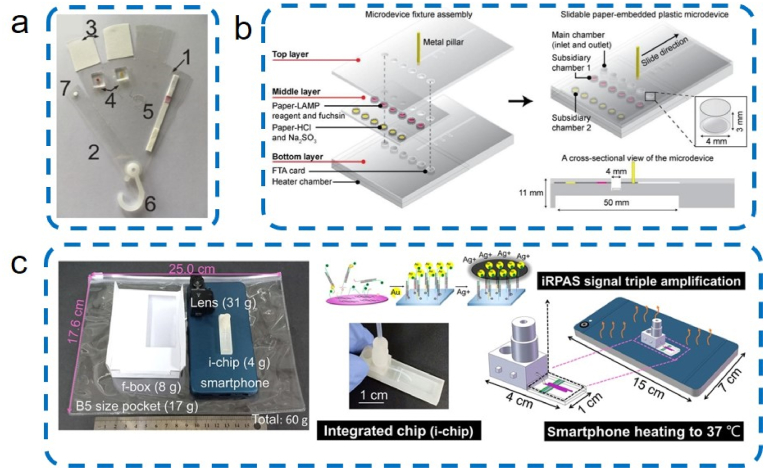
Integrated portable microfluidic chip for nucleic acid detection: (a) paper chip [214]. 1: laminating sheets, 2: adhesive sheets with 1 mm diameter hole, 3: two quantitative filter papers as absorbent pads, 4: two PDMS reservoirs, 5: a LFD strip, 6: a button for chip assembly, 7: a FTA card, Copyright 2019, Elsevier; (b) slidable paper-embedded plastic microfluidic chip [215]. Copyright 2019, Elsevier; (c) ultraportable and versatile point-of-care DNA testing platform [216]. Copyright 2020, AAAS.

Nucleic acids (NAs) detection on microfluidic device can be divided into three sections: NA extraction (eg. silica matrices, cellulose matrices, and magnetic beads), NA amplification (eg. PCR, LAMP, and RCA), and signal readout [213]. Fully-integrated NA detection devices are emerging for POCT, requiring portability, low cost, sensitivity, and rapidness. Chen et al. [214] demonstrated an ultraportable paper-based microfluidic chip using flinders technology associates (FTA) card for on-chip and preloading FTA purification buffer in PDMS reservoirs. Trinh et al. [215] fabricated a slidable paper-embedded plastic microdevice that enables quantitative detection of multiple foodborne pathogens in 75 min (Fig. 10).

Multiple amplification techniques have been developed to further increase sensitivity. Xu et al. [216] created an iRPAS signal triple amplification method through recombinase polymerase amplification (RPA), biotin binding with the streptavidin-conjugated gold nano particles (SA-AuNPs) and silver ion catalytic cascade. Yin et al. [217] reported a sensitive and simple method for multiplex on-site detection. The device utilizes FTA membrane for NA purification and combines RPA and LAMP reaction to export colorimetric signal which can be recognized by the naked eye and a smartphone. Miniaturization of the device is another POCT aspect. Kadimisetty et al. [178] fabricated a 3D printed POC diagnostic device along with static coating of reactor arrays capable of sample to target in variety of body fluids.

These studies greatly improved device simplicity and user-friendliness, but high-speed detection is also significant under emergency situations. Koo et al. [218] realized sample-to-targeted genetic analysis by electrical lysis and nanofluidic manipulation accelerated solid phase RPA on a biochip in 30 min. Li et al. [219] fabricated a SARS-CoV-2 aerosol monitoring system (SIAMs) combining virus RNA extraction, based on chitosan modified quartz filter with tpRPA into an integrated microfluidic box with a time as short as 25 min. Bender et al. [220] presented a paper-based NA testing device that preforms ITP extraction and RPA amplification simultaneously from serum or whole blood to target in less than 20 min. Ouyang et al. [221] developed one-step NA purification using hierarchical selective electrokinetic concentration analyzing ultra-low abundance NAs on an integrated chip in 15 min. These lab-on-chip platforms effectively connects procedures of cell lysis, NA amplification, and signal output, reducing liquid exchange time.

## 4 Concluding remarks and perspectives

Biosensing based on open and closed microfluidics, including fundamentals, liquid control methods, and applications, has been summarized. For open microfluidics, liquids would contact with air or another immiscible liquid, and liquids are mainly manipulated as droplets; for packaged microfluidics, liquids are controlled inside pre-set channels as continuous liquids or droplets inside continuous liquids. The differences like liquid form, chip structure lead to different fundamentals, liquid manipulation methods, and practical applications of open and closed microfluidics, which are systematically summarized. For open microfluidics, fundamentals of wetting phenomena including spreading parameter, contact angles, and contact angle hysteresis are introduced; droplet manipulation methods with external physical fields like electric, magnetic, or optical field are shown; applications including immunoassay, single-cell analysis, biomolecular analysis, drug screening, and medical diagnostics are summarized. For closed microfluidics, fundamentals including related dimensionless number and chip materials are listed; liquid control methods and applications like enzyme-linked immunosorbent assay and nucleic acids detection are also introduced.

Closed microfluidics confine liquids within channels. Although their manufacturing and maintenance are complex and costly, the reduced contamination risk makes them essential for applications requiring stringent sample purity requirements. Conversely, open microfluidics, characterized by their simplicity and accessibility, offer a cost-effective alternative across a spectrum of applications. While exhibiting less precision in liquid control compared to their closed counterparts, these systems find favor in experiments prioritizing operational simplicity and cost-effectiveness. Open microfluidics is ideal for high-throughput and small-volume biological analyses, while closed microfluidics is more suited for stable flow reactions and high-sensitivity analytical domains. Both can be applied in biomedical research, encompassing areas such as immunoassay, nucleic acid analysis, and biomarker detection. Careful assessment of precision requirements, experimental complexities, and cost are required for the determination of the most appropriate microfluidic system.

Although integrated microfluidics for various biological detections has significant potential in a variety of scenarios, it is still in its early stages despite the rapid development of microfluidics. The development of multiple-detection microfluidics devices can be pursued to reduce testing time and expense for varied and high-throughput biological detection.

The detection sensitivity and chip durability for biosensing based on microfluidics are still being developed and enhanced for the practical applications, which is essential for microfluidics used for point-of-care testing. Chip material (polymer or silicon, for example), chip structure (open/closed), liquid manipulation method (passive/active method), reaction, and detection process design should all be taken into account to increase sensitivity and durability.

## CRediT authorship contribution statement

**Tianxin Ge:** Writing – original draft. **Wenxu Hu:** Writing – original draft. **Zilong Zhang:** Writing – original draft. **Xuexue He:** Writing – original draft. **Liqiu Wang:** Supervision. **Xing Han:** Writing – review & editing, Visualization, Supervision, Resources, Investigation. **Zong Dai:** Supervision.

## Declaration of competing interest

The authors declare that they have no known competing financial interests or personal relationships that could have appeared to influence the work reported in this paper.

## Data Availability

Data will be made available on request.
